# Supplementary material for: Successional Shifts in Soil C:N:P stoichiometry and its environmental controls during forest restoration in Huize County, Qujing, Yunnan Province
Source: PLoS One. 2026 Aug 21;21(8):e0356261. doi: 10.1371/journal.pone.0356261 (PMC13496410; doi:10.1371/journal.pone.0356261)
Supplement: Supplementary file 2 [file pone.0356261.s004.zip › Data and Code/RF/evergreen broadleaf forest/figures/importance_C.P.pdf]

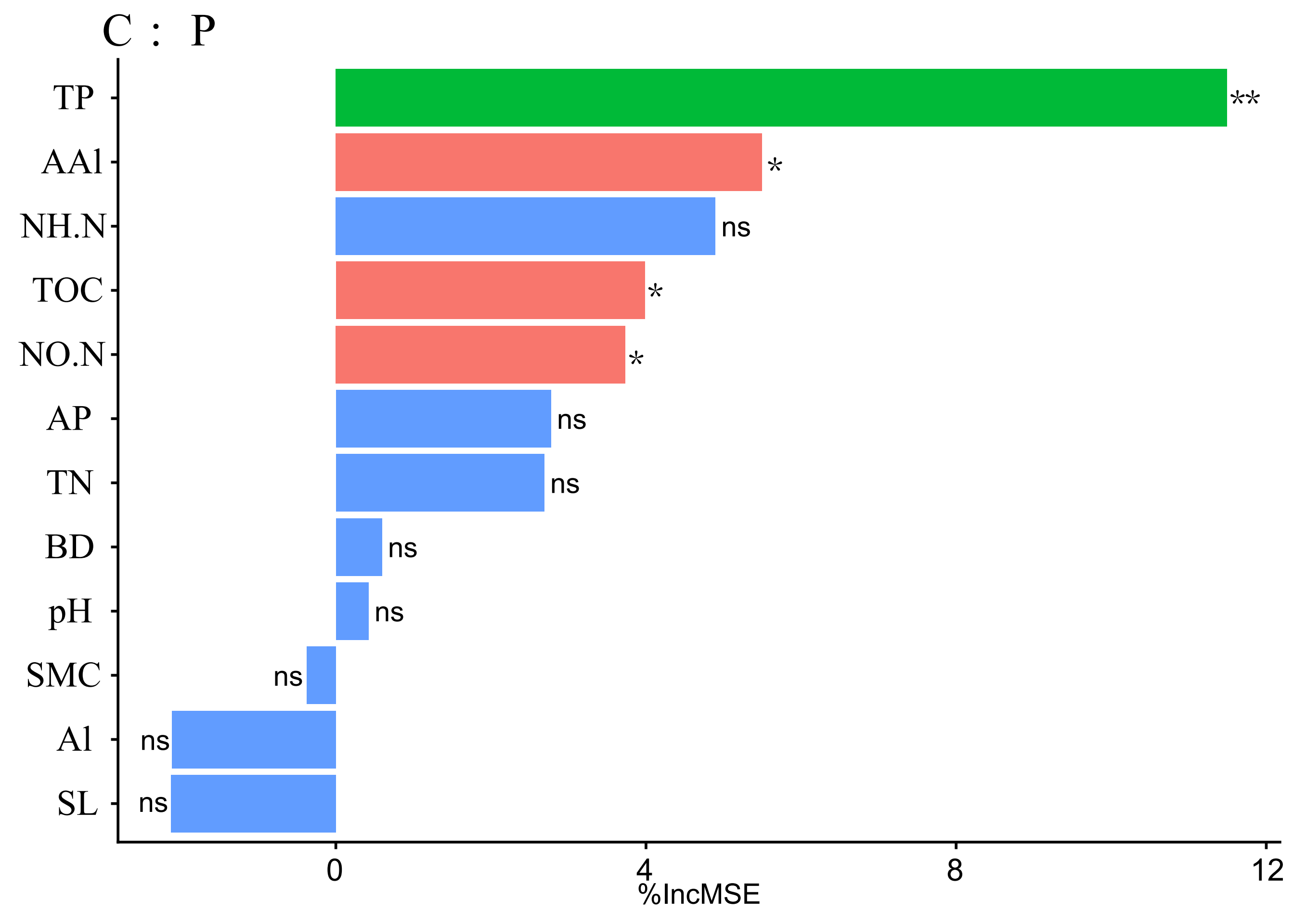

C : P
TP
AAl
NH.N
TOC
NO.N
AP
TN
BD
pH
SMC
Al
SL
**
*
ns
*
*
ns
ns
ns
ns
ns
ns
ns
0
4
8
12
%IncMSE
